# Patient and service factors associated with referral and admission to inpatient rehabilitation after the acute phase of stroke in Australia and Norway

**DOI:** 10.1186/s12913-019-4713-x

**Published:** 2019-11-21

**Authors:** Angela S. Labberton, Mathias Barra, Ole Morten Rønning, Bente Thommessen, Leonid Churilov, Dominique A. Cadilhac, Elizabeth A. Lynch

**Affiliations:** 1 Health Services Research Unit, Akershus University Hospital, PO Box 1000, 1478 Lørenskog, Norway; 2 Institute of Clinical Medicine, University of Oslo, Oslo, Norway; 3 Centre for Connected Care, Oslo University Hospital, Oslo, Norway; 4 Department of Neurology, Akershus University Hospital, Lørenskog, Norway; 5 Department of Medicine (Austin Health), Melbourne Medical School, The University of Melbourne, Heidelberg, Australia; 6 NHMRC Centre of Research Excellence in Stroke Rehabilitation and Brain Recovery, Melbourne and Newcastle, Melbourne, Australia; 7 Stroke Division, The Florey Institute of Neuroscience and Mental Health, University of Melbourne, Heidelberg, Australia; 8 Stroke and Ageing Research, Department of Medicine, School of Clinical Sciences at Monash Health, Monash University, Clayton, Australia; 9 Adelaide Nursing School, University of Adelaide, Adelaide, Australia

**Keywords:** Rehabilitation, Stroke, Referral and consultation, Health services

## Abstract

**Background:**

Unequal access to inpatient rehabilitation after stroke has been reported. We sought to identify and compare patient and service factors associated with referral and admission to an inpatient rehabilitation facility (IRF) after acute hospital care for stroke in two countries with publicly-funded healthcare.

**Methods:**

We compared two cohorts of stroke patients admitted consecutively to eight acute public hospitals in Australia in 2013–2014 (*n* = 553), and to one large university hospital in Norway in 2012–2013 (*n* = 723). Outcomes were: referral to an IRF; admission to an IRF if referred. Logistic regression models were used to identify and compare factors associated with each outcome.

**Results:**

Participants were similar in both cohorts: mean age 73 years, 40–44% female, 12–13% intracerebral haemorrhage, ~ 77% mild stroke (National Institutes of Health Stroke Scale < 8). Services received during the acute admission differed (Australia vs. Norway): stroke unit treatment 82% vs. 97%, physiotherapy 93% vs. 79%, occupational therapy 83% vs. 77%, speech therapy 78% vs. 13%. Proportions referred to an IRF were: 48% (Australia) and 37% (Norway); proportions admitted: 35% (Australia) and 28% (Norway). Factors associated with referral in both countries were: moderately severe stroke, receiving stroke unit treatment or allied health assessments during the acute admission, living in the community, and independent pre-stroke mobility. Directions of associations were mostly congruent; however younger patients were more likely to be referred and admitted in Norway only. Models for admission among patients referred identified few associated factors suggesting that additional factors were important for this stage of the process.

**Conclusions:**

Similar factors were associated with referral to inpatient rehabilitation after acute stroke in both countries, despite differing service provision and access rates. Assuming it is not feasible to provide inpatient rehabilitation to all patients following stroke, the criteria for the selection of candidates need to be understood to address unwanted biases.

## Background

Stroke patients often have persisting deficits requiring complex rehabilitation after the acute phase. The acute hospital length of stay (LOS) is often under 2 weeks for stroke [1], and post-acute rehabilitation is usually provided in other settings: at home, in the community, or in inpatient rehabilitation facilities (IRFs). The latter is the most clearly defined and standardized: IRFs are dedicated facilities or wards, and the rehabilitation is coordinated, interdisciplinary, and in many countries minimum requirements for staffing, therapy types, quantity and duration are specified [2–6]. Post-acute rehabilitation in IRFs is appropriate for patients with complex medical and rehabilitation needs unable to be met in other settings [7].

There is good evidence for the benefits of coordinated, inpatient rehabilitation after stroke: decreased death, dependency and use of institutional care, and greater functional gains [2, 8–11]. However, the selection of patients for post-acute rehabilitation in IRFs (or *inpatient rehabilitation*) has been described as subjective [12], and access varies from country to country, ranging from 13% (Sweden) to 57% (Israel) [1]. Admission to inpatient rehabilitation may depend on factors such as access to and LOS in stroke units (SUs), capacity and funding of IRFs, alternative options for providing rehabilitation, as well as patient characteristics and preferences [13], which may include whether they prefer another type of rehabilitation setting or location, or whether they do not wish to receive rehabilitation at all.

Various authors have reported unequal access to inpatient rehabilitation after stroke [1, 14–17]. Referral from the acute ward to inpatient rehabilitation is an important gate-keeping step: generally referral is a necessary condition for access, because only the referred patients are reviewed by the rehabilitation intake team for consideration for admission [18]. Referral of the appropriate patients is therefore essential to ensure equitable access. While there is a substantial body of evidence about factors associated with admission to IRFs following stroke [14, 19–24], less is known about the essential pre-admission criteria influencing being referred in the first place [16]. Identification of determinants, as well as similarities and differences with regard to referral and admission practices, will be useful if action is required to change the way rehabilitation is organized.

To this end we compared access to inpatient rehabilitation in two countries with universal, publicly-funded health-care. Inpatient rehabilitation was chosen because this type of rehabilitation is the most standardized and well-defined type in stroke guidelines [2], and therefore most comparable across countries [1]. The aim was to identify and compare factors associated with referral, and admission, to an IRF after the acute hospital stay among patients with stroke admitted to public hospitals in Australia and Norway.

## Methods

### Study context

Australia and Norway provide publicly-funded healthcare to all citizens, including IRF admissions for patients with stroke. Private health insurance is supplementary or complementary to public services in both countries, noting that a larger proportion of Australians have private health insurance coverage than in Norway (approximately 56% vs. 9%) [25]. Both countries have had a median LOS for acute stroke admissions of 5 days in recent years [26–28] and post-acute inpatient rehabilitation therefore generally takes place in a separate ward or hospital providing IRF care. There is no equivalent to skilled nursing facilities in either country. In Australia, referred patients are usually assessed in person by an IRF-representative before a decision is made regarding acceptance [18]. The IRF-representative is usually a rehabilitation physician who works at the IRF to which the patient has been referred. In Norway, referred patients are discussed by the IRF during multidisciplinary intake meetings and only reviewed by an IRF-representative in person in uncertain cases.

### Participants

We used data from two pre-existing cohorts of patients with stroke in Australia and Norway. Consecutively admitted adult patients with a stroke diagnosis were included, defined by an *International Classification of Diseases-10th revision* discharge code of I61, I62.9, I63, or I64. Exclusion criteria were: in-hospital death, palliative treatment or coma, no stroke symptoms on admission, and hospital transfer for acute treatment.

The Australian data comprise two retrospective medical record audits from 10 acute public hospitals in South Australia and New South Wales [29]. Two hospitals were located in rural areas without SUs, admitting ~ 30 stroke cases annually, and were excluded from the current study. The eight remaining hospitals were located in metropolitan areas, had SUs, and admitted a mean of 450 (range 130–800) stroke cases annually. Consecutive stroke admissions between 1st October 2012 – 1st August 2014 were audited. Each hospital was audited twice during the study period, with a maximum of 45 records per site per audit. Data from additional cases from one participating hospital were available and were included in the present study. One hospital had a co-located IRF while the others referred patients to separately located public and private IRFs.

The Norwegian data are from the Norwegian Stroke – Paths of Treatment (NOR-SPOT) cohort [30], prospectively collected to investigate health service delivery to stroke patients. Consecutive stroke admissions to Akershus University Hospital were recorded between 15th February 2012 – 15th March 2013. The hospital is situated in greater metropolitan Oslo and is the only hospital in the region admitting patients with acute stroke (~ 850 cases annually). It has a co-located IRF and only ~ 1% of patients are referred to private IRFs sub-contracted to the regional health authority.

### Outcomes and measures

The primary outcome was referral to an IRF for inpatient rehabilitation. The secondary outcome was admission to an IRF, among referred. In the Australian-cohort a patient was considered to have been referred if an assessment by an IRF-representative was documented, or if the patient was admitted to an IRF (referral forms were often not filed in the paper-based medical records). In the Norwegian-cohort referrals were documented in, and extracted from, the electronic medical records. Admission to an IRF was documented in the medical records in both countries.

Pre-specified factors, previously shown to be associated with accessing rehabilitation were recorded [12, 18, 21, 22, 31, 32]. Patient-related factors were: age, sex, pre-stroke place of living (community alone, community with others, nursing home), and pre-stroke mobility (requiring human assistance or not). Disease-related factors were: stroke severity and type (intracerebral hemorrhage or ischemic). Stroke severity was determined by National Institutes of Health Stroke Scale (NIHSS) [33] scores within 24 h of admission. Where a prospective NIHSS score was unavailable, author EAL or a trained assistant (Australia), or ASL (Norway), scored patients retrospectively using admission records and, in Norway, a validated algorithm [34]. Service-related factors were: receiving SU treatment and documented assessments by allied health staff (physiotherapy, occupational therapy, speech therapy) during the acute admission.

Additional relevant factors [12, 23, 31] were available in the Norwegian-cohort only. These were documentation during the acute admission of: history of cognitive impairment or dementia, post-stroke Mini Mental State Examination (MMSE) score [35], and post-stroke Barthel Index (BI) [36] or modified Rankin Scale (mRS) score [37]. The BI and mRS are highly correlated [38], and patients were classified as being independent by either BI score ≥ 85 or mRS score ≤ 2. These cutoffs are shown to be comparable and commonly denote “good outcome” [38, 39].

### Statistical methods

Independent samples *t*-tests were used for between-cohort differences in continuous variables and chi-squared tests for categorical variables. Associations between the factors and each outcome (IRF referral; and IRF admission if referred) were assessed with logistic regression. Due to data privacy regulations we were unable to pool data to analyse a common dataset. Therefore, we implemented the same analysis plan with the same pre-specified common set of factors in each dataset, separately. Unadjusted odds ratios (ORs) and adjusted odds ratios (aORs) are presented. Multivariable models were adjusted for the same three potentially confounding factors (age, sex, NIHSS score) to provide aORs for each factor in each country. NIHSS was entered as a continuous variable and a quadratic term was added to allow for an expected non-linear peak around moderately severe strokes. Multi-level random effects logistic models were fitted to the Australian data to account for within-hospital clustering. Clustering was assessed via the intraclass correlation coefficient (ICC). For the primary outcome (referral), we also estimated standardized coefficients on the multivariable models. These provide a log-odds per standard deviation interpretation of the coefficients [40], and facilitate comparison of the relative effect sizes across different factors and datasets. Between-country difference for regression coefficients were assessed with the z-test [41]. Finally, we fitted models containing all candidate factors to each dataset, and for each outcome, to estimate how well the outcomes could be predicted when all factors were considered, measured by the area under the receiver operating characteristic curve (AUROC) [42]. Collinearity between independent variables was assessed via variance inflation factors and condition numbers. Analyses were performed using the statistical software StataIC (StataCorp, College Station, TX) and R (R Foundation for Statistical Computing, Vienna, Austria), and significance level set at 0.05.

## Results

**Fig. 1:**
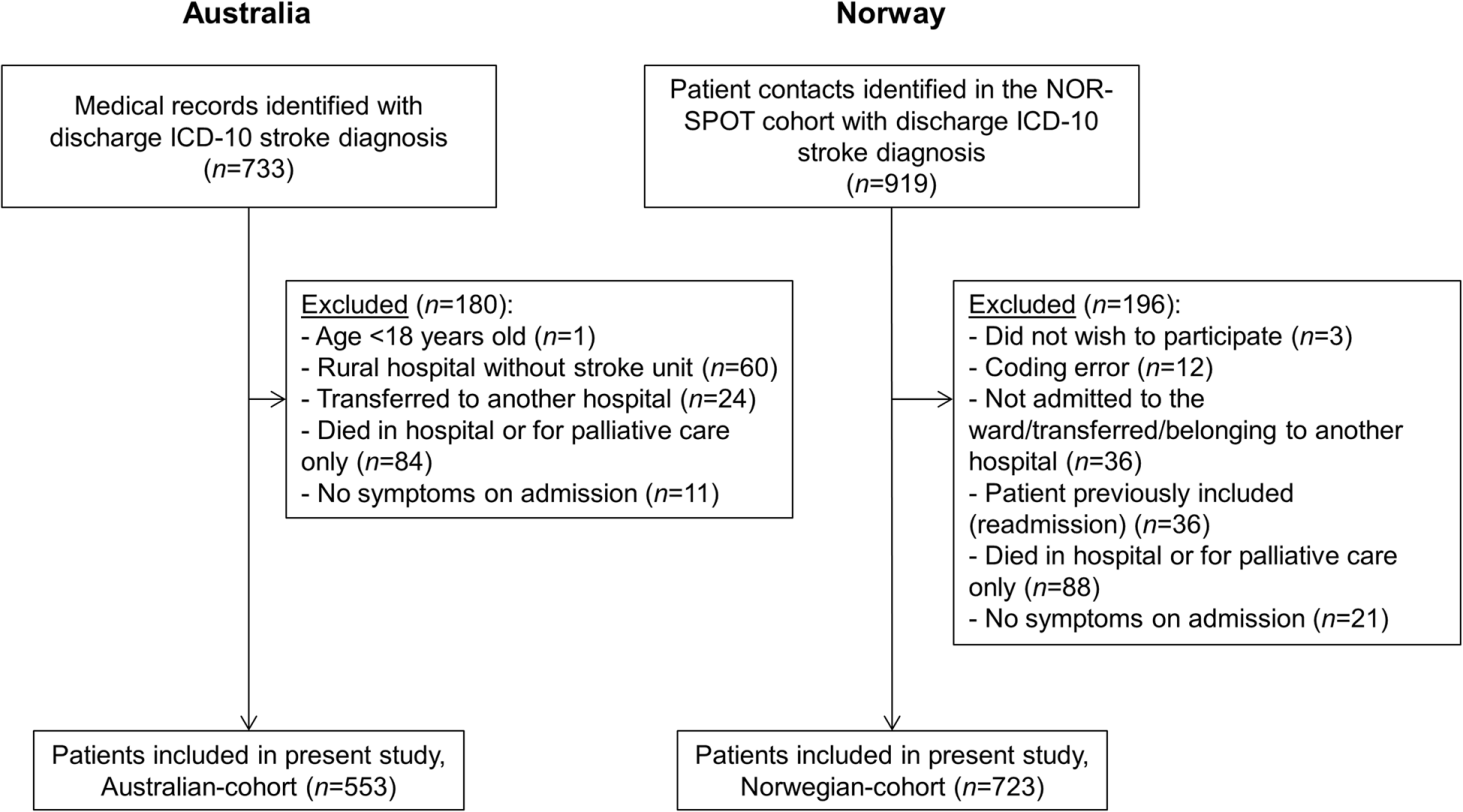
Inclusion flowchart. ICD-10 = International Classification of Diseases-10th revision; NOR-SPOT = Norwegian Stroke – Paths of Treatment.

In total 1276 patients were included: 553 from Australia, 723 from Norway (Fig. 1). The cohorts were similar for most patient and clinical characteristics (Table 1): mean age 73 years, females (Australia 40% vs. 44%, *p* = .19), 4% dependently mobile pre-stroke, 12–13% intracerebral haemorrhage, and ~ 77% mild strokes (NIHSS< 8).

**Table 1:** Description of the cohorts

Patient factors	Australia (*n* = 553)	Norway (*n* = 723)	*p*	Missing ^a^ *n*
Age in years, mean (SD)	73.2 (14.2)	72.8 (13.9)	.61	2
Female sex	222 (40.3)	318 (44.0)	.19	2
Pre-morbid place of living:			<.001	1
Community with others	374 (67.8)	443 (61.3)		
Community alone	124 (22.5)	240 (33.2)		
Nursing home	54 (9.8)	40 (5.5)		
Dependent pre-stroke mobility	24 (4.3)	26 (3.6)	.50	0
Disease factors				
Intracerebral hemorrhage	64 (11.6)	96 (13.3)	.36	0
NIHSS:				0
Mean (SD)	5.1 (5.1)	5.3 (5.9)	.53	
Median (Q1 – Q3)	4 (2–7)	3 (1–7)		
Mild (< 8)	423 (76.5)	562 (77.7)	.12	
Moderate (8–16)	103 (18.6)	111 (15.4)		
Severe (> 16)	27 (4.9)	50 (6.9)		
Service factors				
Received stroke unit treatment	451 (82.0)	703 (97.2)	<.001	3
Received physiotherapy	497 (92.9)	568 (78.6)	<.001	18
Received occupational therapy	437 (82.5)	554 (76.6)	.01	23
Received speech therapy	413 (77.8)	96 (13.3)	<.001	22
Any allied health input	518 (96.8)	639 (88.4)	<.001	18

Values expressed as *n* (%) unless otherwise specified

SD = standard deviation; Q1 = 25th percentile; Q3 = 75th percentile

^a^ Missing values are in the Australian cohort only

Service-characteristics differed: more patients in Australia received physiotherapy (93% vs. Norway 79%, *p* < .001), occupational therapy (83% vs. Norway 77%, *p* = .01), and speech therapy (78% vs. Norway 13%, *p* < .001) during the acute admission, whereas almost all in Norway received SU treatment (82% vs. Norway 97%, *p* < .001).

Additional factors available in the Norwegian-cohort were: 72/723 (10%) had a history of cognitive impairment or dementia, and post-stroke assessments showed median MMSE score 25 (interquartile range 20–28; *n* = 430), 333/606 (55%) were considered independent, and 287/375 (77%) were continent of urine.

### Proportions referred and admitted to inpatient rehabilitation

In Australia, 264 (47.7% [95% confidence interval: 43.5, 52.0]) were referred to inpatient rehabilitation and 191 (34.5% [30.6, 38.7]) admitted. In Norway, 267 (36.9% [33.4, 40.6]) were referred and 200 (27.7% [24.4, 31.1]) were admitted.

### Factors associated with referral to inpatient rehabilitation

**Fig. 2:**
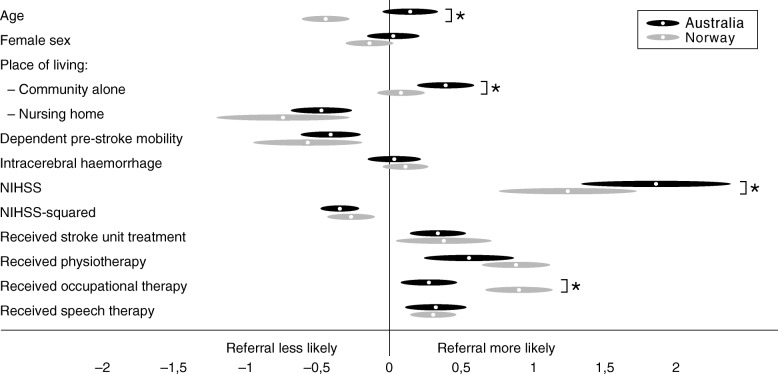
Standardized coefficients and their 95% confidence intervals for referral to inpatient rehabilitation. Graphical representation of the standardized coefficients for the multivariable models (adjusted for age, sex, NIHSS). See Additional file 1: Table S1 for the raw and standardized coefficients. NIHSS=National Institutes of Health Stroke Scale. * *p* < 0.05, z-test.

The odds (adjusted for age, sex and NIHSS score) of referral were increased in both countries, and by similar amounts, for patients who received physiotherapy (aOR Australia 8.62 [2.53, 29.28]; Norway 8.47 [4.74, 15.12]) and speech therapy (aOR Australia 2.14 [1.29, 3.57]; Norway 2.41 [1.51, 3.86]) (Table 2). Occupational therapy appeared to have greater importance in Norway (aOR Australia 2.03 [1.22, 3.39]; Norway 8.31 [4.75, 14.54], *p* < .001) (Fig. 2, Additional file 1: Table S1). SU treatment was also positively associated (aOR Australia 2.38 [1.43, 3.94]; Norway 9.83 [1.29, 75.10]), noting the large confidence interval for Norway because almost all patients received SU treatment. Factors similarly, and negatively, associated with referral in both countries were admission from nursing home and dependent pre-stroke mobility.

**Table 2:** Factors associated with referral to inpatient rehabilitation

Factor	Australia (n = 553)		Norway (n = 723)	
	OR (95% CI)	aOR (95% CI)	OR (95% CI)	aOR (95% CI)
Age	1.02 (1.01, 1.04)	1.01 (1.00, 1.02)	0.97 (0.96, 0.98)	0.97 (0.96, 0.98)
Female sex	1.16 (0.83, 1.64)	1.05 (0.72, 1.52)	0.72 (0.53, 0.98)	0.74 (0.53, 1.03)
Place of living:				
Community with others	Reference		Reference	
Community alone	2.62 (1.71, 4.01)	2.52 (1.56, 4.08)	0.99 (0.72, 1.36)	1.17 (0.83, 1.67)
Nursing home	0.59 (0.32, 1.09)	0.20 (0.10, 0.40)	0.04 (0.01, 0.29)	0.04 (0.01, 0.29)
Dependent pre-stroke mobility	0.35 (0.14, 0.90)	0.13 (0.05, 0.36)	0.06 (0.01, 0.48)	0.05 (0.01, 0.40)
Intracerebral haemorrhage	0.96 (0.57, 1.62)	1.09 (0.61, 1.95)	1.61 (1.04, 2.48)	1.37 (0.86, 2.17)
NIHSS	1.13 (1.09, 1.18)	1.44 (1.30, 1.60)	1.02 (1.00, 1.05)	1.23 (1.14, 1.34)
NIHSS-squared	*–*	0.99 (0.98, 0.99)	*–*	0.99 (0.99, 0.996)
Received stroke unit treatment	1.97 (1.25, 3.11)	2.38 (1.43, 3.94)	11.57 (1.54, 86.89)	9.83 (1.29, 75.10)
Received physiotherapy	11.90 (3.61, 39.21)	8.62 (2.53, 29.28)	6.88 (4.00, 11.84)	8.47 (4.74, 15.12)
Received occupational therapy	2.32 (1.44, 3.74)	2.03 (1.22, 3.39)	7.92 (4.61, 13.61)	8.31 (4.75, 14.54)
Received speech therapy	3.85 (2.41, 6.13)	2.14 (1.29, 3.57)	2.76 (1.78, 4.28)	2.41 (1.51, 3.86)

Univariable and multivariable analyses for factors associated with referral within each cohort. Multivariable analyses (aOR) adjusted for age, sex, and NIHSS (continuous and squared). Australian data adjusted for clustering within hospitals

OR = odds ratio; 95% CI = 95% confidence interval; aOR = adjusted OR; NIHSS=National Institutes of Health Stroke Scale

In both countries, higher admission NIHSS was associated with referral and the negative coefficients on the NIHSS-squared terms indicated a negative quadratic pattern for NIHSS scores, ie. patients with moderate NIHSS scores were most likely to be referred, peaking at approximately NIHSS 13 in both countries. The countries differed on how age affected referral, with younger patients more likely to be referred in Norway only.

The Norway-only factors (Table 3) indicated that patients with a history of cognitive impairment or dementia, and those considered independent on post-stroke assessments, were less likely to be referred.

**Table 3:** Associations for additional Norway-only factors

Factor	Referral (n = 723) ^a^		Admission (*n* = 267) ^b^	
	OR (95% CI)	aOR (95% CI)	OR (95% CI)	aOR (95% CI)
History of cognitive impairment or dementia	**0.14 (0.06, 0.32)**	**0.13 (0.05, 0.30)**	0.32 (0.06, 1.65)	0.48 (0.09, 2.48)
MMSE score	0.97 (0.94, 1.00)	0.97 (0.93, 1.01)	**1.08 (1.02, 1.14)**	**1.07 (1.01, 1.15)**
Independent post-stroke ^c^	**0.50 (0.36, 0.70)**	**0.37 (0.24, 0.57)**	0.98 (0.54, 1.78)	1.05 (0.53, 2.07)
Continent of urine post-stroke	0.64 (0.40, 1.04)	0.81 (0.46, 1.42)	1.90 (0.93, 3.86)	1.77 (0.77, 4.08)

Univariable and multivariable analyses for factors associated with referral (among all patients) and admission (among referred patients) to inpatient rehabilitation. Multivariable analyses (aOR) adjusted for age, sex, NIHSS (continuous and squared)

OR = odds ratio; 95% CI = 95% confidence interval; aOR = adjusted OR; MMSE = Mini Mental State Examination; NIHSS=National Institutes of Health Stroke Scale

^a^ Exceptions: MMSE score (*n* = 430); Independent post-stroke (*n* = 606); continent of urine post-stroke (*n* = 375)

^b^ Exceptions: MMSE score (*n* = 198); Independent post-stroke (*n* = 234); continent of urine post-stroke (*n* = 177)

^c^ Independent = Barthel Index score ≥ 85 or modified Rankin Scale score ≤ 2

Models containing all candidate factors predicted referral equally well within their respective datasets and with fair discrimination (AUROC Australia 0.79 [0.76, 0.83]; Norway 0.79 [0.75, 0.82]). There was no between-hospital variation in the Australian-cohort (ICC = 0.00 [0.00, 0.00]).

### Factors associated with admission to inpatient rehabilitation among patients referred

There were few associations identified for admission among the referred (Table 4). Associated factors in Australia were dependent pre-stroke mobility and receiving speech therapy. In the Norwegian-cohort, three factors (admission from nursing home; dependent pre-stroke mobility; not receiving SU treatment) perfectly predicted *not* being admitted and estimates could not be calculated. Younger age was positively associated with admission in Norway.

**Table 4:** Factors associated with admission to inpatient rehabilitation

Factor	Australia (*n* = 264)		Norway (n = 267)	
	OR (95% CI)	aOR (95% CI)	OR (95% CI)	aOR (95% CI)
Age	0.98 (0.96, 1.00)	0.98 (0.96, 1.01)	**0.96 (0.93, 0.98)**	**0.95 (0.93, 0.98)**
Female sex	0.61 (0.35, 1.08)	0.68 (0.38, 1.23)	1.19 (0.67, 2.12)	1.33 (0.72, 2.44)
Place of living:				
Community with others	Reference		Reference	
Community alone	1.12 (0.60, 2.09)	1.26 (0.65, 2.44)	0.71 (0.40, 1.26)	0.81 (0.44, 1.51)
Nursing home	0.87 (0.27, 2.82)	1.01 (0.31, 3.38)	a	a
Dependent pre-stroke mobility	**0.09 (0.01, 0.80)**	**0.09 (0.01, 0.89)**	a	a
Intracerebral haemorrhage	0.86 (0.36, 2.03)	0.85 (0.35, 2.05)	1.04 (0.50, 2.19)	0.83 (0.38, 1.83)
NIHSS	0.95 (0.90, 1.01)	0.98 (0.83, 1.16)	1.05 (0.99, 1.11)	1.12 (0.96, 1.30)
NIHSS-squared	–	1.00 (0.99, 1.01)	–	1.00 (0.99, 1.00)
Received stroke unit treatment	1.18 (0.51, 2.70)	1.02 (0.43, 2.46)	a	a
Received physiotherapy	6.68 (0.54, 81.93)	11.69 (0.81, 168.28)	0.99 (0.31, 3.20)	1.31 (0.38, 4.56)
Received occupational therapy	1.12 (0.46, 2.72)	1.46 (0.58, 3.67)	0.99 (0.31, 3.20)	1.86 (0.52, 6.68)
Received speech therapy	1.84 (0.78, 4.36)	**2.70 (1.05, 6.94)**	1.48 (0.71, 3.06)	1.24 (0.57, 2.71)

Univariable and multivariable analyses for factors associated with admission within each cohort, among the patients referred. Multivariable analyses (aOR) adjusted for age, sex, and NIHSS (continuous and squared)

OR = odds ratio; 95% CI = 95% confidence interval; aOR = adjusted OR; NIHSS=National Institutes of Health Stroke Scale

^a^ Unable to be estimated due to small or empty cells

In the Norwegian-cohort, a higher MMSE score was associated with admission, for the sub-set who were tested post-stroke and referred to inpatient rehabilitation (*n* = 198) (Table 3).

Models containing all candidate factors predicted admission equally poorly within their respective datasets (AUROC Australia 0.68 [0.60, 0.75]; Norway 0.68 [0.61, 0.75]). There was 10% between-hospital variation in the Australian-cohort (ICC = 0.10 [0.02, 0.34]).

## Discussion

We have identified factors associated with referral, and admission, to post-acute inpatient rehabilitation for stroke in Australia and Norway. Acute service provision and IRF access rates differed. Nevertheless, clinicians appeared to consider, and respond similarly to, many of the same factors when deciding which patients to refer. In both countries moderate stroke severity and receiving SU treatment or allied health assessments were positively associated with referral, while admission from nursing home and dependent pre-stroke mobility were negatively associated. Few factors were identified as being associated with admission among patients referred, suggesting that additional unmeasured factors are relevant for this stage of the decision-making process.

Other authors have similarly observed that stroke severity influences patient selection for rehabilitation [15, 19]. In the present study, patients with milder strokes were less likely to be referred to IRFs compared to moderate strokes. They may have instead been referred to early supported discharge (ESD) services. However, although recommended in both countries [3, 6], ESD is limited: only 11% of Australian hospitals in 2017 and one hospital in the largest health region in Norway in 2018 reported access to ESD services [43, 44]. Furthermore, patients with severe strokes may miss out on coordinated rehabilitation completely. Inpatient rehabilitation can also benefit these patients [8, 45], however, the rehabilitation is resource-intensive, and this group is often not prioritized, possibly due to perceived limited rehabilitation potential [45]. Similarly, patients admitted from nursing homes or those with dependent pre-stroke mobility were unlikely to be referred. Selection of patients based on prior independence has been previously described [12, 15, 31] and guidelines in some countries or facilities [1, 20], including in Norway [46], afford weight to whether the patient is expected to return to community living. We observed that younger patients were more likely to be both referred and admitted to IRFs in the Norwegian-cohort, further evidence for the prioritization of pre-morbidly independent community-dwellers [22].

Receiving allied health assessment during the acute admission may be an indication of functional impairment and need for rehabilitation, and was associated with IRF referral in both countries. However, proportions assessed by allied health staff were smaller in the Norwegian-cohort, reflecting contrasting use of resources. In Norway, although all stroke patients are discussed during multidisciplinary team meetings, only selected patients are referred for in-person assessment or treatment by therapists. In contrast, in Australia it is specifically recommended that stroke patients should be assessed by a physiotherapist within 24–48 h of admission [47], and that rehabilitation therapy should commence within 48 h of initial assessment [47]. Assessments by other allied health professionals are audited in the biennial Australian national audit, highlighting the tacit recommendation in Australia that all patients admitted with stroke should be assessed by allied health professionals. We observed that the proportions of patients receiving speech therapy assessments were especially different (Australia 78% vs. Norway 13%), which is likely because many Australian SUs have blanket referral to physiotherapy, occupational therapy and speech therapy. Further, patients in Australia are often referred to speech therapists for swallow assessments [13], whereas in Norway the nursing staff perform more detailed testing resulting in fewer referrals to therapists. Our data do not distinguish between therapy assessments for the purpose of screening versus for active therapy.

Approximately one-quarter of referred patients were not admitted to IRFs in both countries. Admission to IRFs among the patients referred was not able to be predicted well in our analyses when all factors were considered (low AUROC). This suggests that most of the patient selection had already occurred at the referral stage, or that other unmeasured and harder-to-quantify factors such as motivation, mood, IRF capacity, expected prognosis and patient/family preferences are important for selecting between the referred patients. Alternatively, our findings may indicate that patient selection for IRF admission is inconsistent and subject to individual variation [12, 48]. In an effort to provide consistent rehabilitation assessments and guide referrals, a nationally endorsed assessment tool was introduced in Australia in 2012 [3]. Similar initiatives are underway in Norway, including evaluating the scope and quality of post-acute rehabilitation [26]. The process and criteria used to determine which referred patients are admitted to IRFs needs further investigation.

The proportion of patients admitted to IRFs was greater in Australia (35%) than in Norway (28%). Our findings for the Australian-cohort were slightly less than in another Australian study (40%) [15] but similar to a Canadian study in which approximately 37% of stroke survivors were candidates for inpatient rehabilitation [49]. However, our proportions are greater than the respective national estimates due to the study exclusions. Australian national data estimated that 28% of non-palliative stroke survivors accessed IRFs in 2014–15 [32], versus only 14% of Norwegian stroke survivors in 2017 (a further 12% received rehabilitation as inpatients in non-specialized municipal centers, primarily nursing homes) [26]. Therefore, Norway appears to have one of the lowest rates of coordinated inpatient rehabilitation for stroke survivors, comparable to Sweden (13%) [1]. In contrast, access to SU treatment in Norway is among the highest with 94% coverage nationally in 2017 (versus 69% in Australia) [26, 43]. SU treatment was positively associated with referral in our study, in keeping with previous observations that patients treated on SUs more often accessed rehabilitation [32, 50].

Comparisons of health services between countries can be challenging, and observed differences in patient outcomes and access may be heavily influenced by differences in financial models, availability of resources, and clinical guidelines or traditions [51, 52]. In this study, we have compared two countries with similar characteristics: high-income countries with universal health care where private health insurance is supplementary [25]. The respective national stroke guidelines are similar [7, 13] and the model of SUs providing early rehabilitation with subsequent referral to an IRF for patients requiring ongoing inpatient rehabilitation is the most common model. This is supported by the same, relatively short, median length of stay in the acute hospital for stroke of 5 days [26–28]. Furthermore, neither country have skilled nursing facilities, and ESD services are not widely used [43, 44]. While admission to IRFs may be more influenced by external system factors [52], it would appear that referral practices are less so, given the surprisingly similar referral decision-making between the countries. This study is one of few to investigate referral practices to inpatient rehabilitation, and also to make international comparisons. Studies designed to examine associations with admission to IRFs only among the patients already referred may fail to detect all relevant factors.

### Study limitations

Data privacy regulations prevented the pooling of Australian and Norwegian datasets, and legislation is increasingly strict. However, this should not prevent the comparison of services across countries, and here we have shown a possible solution: standardized coefficients which estimate the importance of the factors on the outcome relative to the underlying variation, and thus allow interpretable comparisons across datasets.

The Australian data represent eight acute care hospitals with SUs situated in metropolitan areas across two states. We did not include two hospitals located in rural areas and without SUs to improve comparability with Norway. The Norwegian data represents only one hospital, however the catchment population is large and diverse and represents almost 10% of Norway’s population. The hospital’s access rate for inpatient rehabilitation is high for Norway; however, this may not affect patient selection. We identified several common factors between our cohorts despite differing access rates, and referral was not affected by between-hospital variation in the Australian cohort. Conversely, it may have some impact on the outcome admission, where the Australian data showed 10% between-hospital variation, similar to a previous study (12%) [22].

We have not been able to compare the acute LOS of the hospitals included in this study, however all hospitals included provided early stroke rehabilitation only, and referred patients on to IRFs for further inpatient rehabilitation if needed. We had no, or incomplete, data on several factors previously shown to be associated with accessing inpatient rehabilitation such as pre- and post-stroke cognition and detailed post-stroke functional status, and patient/family preferences could not be accounted for. We do not know if patients accessed inpatient rehabilitation later or independent of the public hospital (eg. via their general practitioner), however we do not expect this to encompass many patients as the majority are referred directly from the acute hospital. Lastly, we do not have information on patients’ insurance status or whether they accessed rehabilitation as a private or public patient, which may be of relevance particularly in Australia.

## Conclusion

There were remarkable similarities in patient selection for referral to post-acute inpatient rehabilitation between the two countries with publicly-funded healthcare. Moderately severe stroke and receiving SU treatment and allied health assessments were positively associated with referral, while patients with pre-existing dependencies were less likely to be either referred or admitted. The capacity to provide inpatient rehabilitation to all patients with stroke may not be feasible given constrained resources. A greater understanding of the criteria being used when referring and admitting patients may identify selection biases that could be addressed; an important issue to ensure equitable use of rehabilitation resources.

## Supplementary information

**Additional file 1: Table S1.** Raw regression coefficients and standardized coefficients for the multivariable models for referral to inpatient rehabilitation.

## Data Availability

The datasets analysed during the current study are not publicly available due to privacy regulations but are available from the corresponding author on reasonable request.

## Abbreviations

95% CI: 95% confidence interval
aOR: adjusted odds ratio
AUROC: area under the receiver operating characteristic curve
BI: Barthel Index
ESD: early supported discharge
ICC: intraclass correlation coefficient
IRF: inpatient rehabilitation facility
LOS: length of stay
MMSE: Mini Mental State Examination
mRS: modified Rankin Scale
NIHSS: National Institutes of Health Stroke Scale
NOR-SPOT: Norwegian Stroke – Paths of Treatment
OR: odds ratio
Q1: 25th percentile
Q3: 75th percentile
SD: standard deviation
SE: standard error
SU: stroke unit
