# Supplementary material for: Patient and service factors associated with referral and admission to inpatient rehabilitation after the acute phase of stroke in Australia and Norway
Source: BMC Health Serv Res. 2019 Nov 21;19:871. doi: 10.1186/s12913-019-4713-x (PMC6873491; doi:10.1186/s12913-019-4713-x)
Supplement: Supplementary file 1 — Additional file 1: Table S1. Raw regression coefficients and standardized coefficients for the multivariable models for referral to inpatient rehabilitation. [file 12913_2019_4713_MOESM1_ESM.docx]

# SUPPLEMENTAL MATERIAL

**Additional file 1: Table S1** Raw regression coefficients and standardized coefficients for the multivariable models for referral to inpatient rehabilitation

| **Factor** | **Australia (*n*=553)** | | **Norway (*n*=723)** | | ***p^*^*** |
| --- | --- | --- | --- | --- | --- |
|  | **Raw regression coefficient (SE)** | **Standardized coefficient (SE)** | **Raw regression coefficient (SE)** | **Standardized coefficient (SE)** |  |
| Age | 0.01 (0.01) | 0.13 (0.10) | -0.03 (0.01) | -0.45 (0.08) | <.001 |
| Female sex | 0.05 (0.19) | 0.02 (0.09) | -0.30 (0.17) | -0.15 (0.08) | .18 |
| Place of living: |  |  |  |  |  |
| - Community with others | Ref. | Ref. | Ref. | Ref. |  |
| - Community alone | 0.93 (0.24) | 0.39 (0.10) | 0.16 (0.18) | 0.08 (0.08) | .01 |
| - Nursing home | -1.62 (0.36) | -0.48 (0.11) | -3.28 (1.03) | -0.75 (0.24) | .13 |
| Dependent pre-stroke mobility | -2.05 (0.52) | -0.42 (0.11) | -2.94 (1.04) | -0.55 (0.19) | .44 |
| Intracerebral haemorrhage | 0.09 (0.29) | 0.03 (0.09) | 0.31 (0.24) | 0.11 (0.08) | .55 |
| NIHSS | 0.37 (0.05) | 1.86 (0.27) | 0.21 (0.04) | 1.24 (0.25) | .02 |
| NIHSS-squared | -0.01 (0.003) | -0.35 (0.07) | -0.01 (0.42) | -0.28 (0.07) | .07 |
| Received stroke unit treatment | 0.87 (0.26) | 0.33 (0.10) | 2.29 (1.04) | 0.38 (0.17) | .18 |
| Received physiotherapy | 2.15 (0.62) | 0.55 (0.16) | 2.14 (0.30) | 0.88 (0.12) | .98 |
| Received occupational therapy | 0.71 (0.26) | 0.27 (0.10) | 2.12 (0.29) | 0.90 (0.12) | <.001 |
| Received speech therapy | 0.76 (0.26) | 0.32 (0.11) | 0.88 (0.24) | 0.30 (0.08) | .74 |

Raw regression coefficients adjusted for age, sex, NIHSS (continuous and squared). The standardized coefficients from this table are shown graphically in Figure 2.

SE=standard error; NIHSS=National Institutes of Health Stroke Scale

* Between-country difference for regression coefficients, z-test
